# Leprosy review

**DOI:** 10.4102/safp.v63i1.5311

**Published:** 2021-10-29

**Authors:** Lehlohonolo Makhakhe

**Affiliations:** 1 Department of Dermatology, Faculty of Health Science, University of the Free State, Bloemfontein, South Africa; 2 The South African Institute of Dermatology, Bloemfontein, South Africa

**Keywords:** leprosy, Hansen’s disease, quality of life, self-esteem, HIV, TB

## Abstract

Leprosy (Hansen’s disease) can affect multiple organs and body structures. Skin signs are typically observed in the early phase of the disease, hence being the first identifiable signs to propel clinical suspicion. Leprosy predominantly affects the skin and peripheral nerves. The disease has been documented many centuries preceding the biblical era. Over many decades, the classification of Hansen’s disease has changed as modern medical science evolved. Patients with leprosy are usually subjected to discrimination, rejection from society and can suffer from social stigma, poor quality of life (QoL), low self-esteem and permanent disfigurements. Studies have shown that leprosy has a significant negative impact on the patients’ QoL. Leprosy is often not suspected by practicing clinicians because it is no longer emphasised in the medical curricula. In modern years, attention has gradually shifted from leprosy to tuberculosis (TB) and human immunodeficiency virus (HIV).

## Introduction

Hansen’s disease is caused by an obligate intracellular organism called *Mycobacterium leprae*. The disease spectrum ranges between two poles, namely tuberculoid and lepromatous, depending on the patient’s immunological response to *M. leprae*.^1^

Before the advent of antimicrobial therapy, patients confirmed with the disease were usually secluded from household contacts and communities at large, in fear of this condition being communicable.

In 2005, the World Health Organization (WHO) reported that leprosy was eliminated as a world public health problem. However, new cases are still seen to this day. Globally, to date, an average of 250 000 new patients are reported annually.^2^ Incidence and prevalence of this condition differs considerably per country, noting that developing countries bear the biggest brunt of both new cases and that of patient on treatment.

Of concern is that little attention is paid to leprosy in the medical curriculum, this has been found to be the case in dermatology, rheumatology and neurology disciplines. As a result of the inadequate medical training, studies have found that clinical suspicion and subsequent diagnosis of leprosy is often delayed, resulting in permanent and debilitating complications. Lack of awareness is one of the main reasons for missing the diagnosis, even at a specialist level.^2^

## Discussion

Hansen’s disease occurs in all ages, in endemic countries such as Brazil, Nepal, India and Indonesia, cases typically present before the age of 35. The latency period between *M. leprae* exposure and overt clinical skin and nerve signs can range from 5 to 10 years depending on the type of leprosy.^3^

Over 80% of persons exposed to the bacterium *M. leprae* will likely resist the inoculation and infection. An estimated 17% of household contacts of individuals infected with leprosy are *M. leprae* polymerase chain reaction (PCR) positive on skin swabs and 4% on nasal swabs. Leprosy has a slight predilection for male adult patients (1.5:1) and monozygotic twins (60% – 85%).^1^

The mode of transmission is documented as ranging from tattooing, household contact and exposure to armadillo. Household contacts represent up to 28% of new cases, they are 4–10 times at risk of infection in the long term.^3^

Because of the long latency period, it is possible for infected persons to only have evidence of the disease from nasal and skin swabs with no overt clinical signs. Hansen’s disease causes pathology in humans and animals. To date, the causative organism, *M. leprae* has not been cultured *in vitro*. It grows best in temperatures of 30 degrees celsius or less, this explains its predilection to the feet, elbows, ears and testicles (in male) whilst sparing the midline and scalp.^1^

Table 1 gives latest available statistics demonstrating incidence of leprosy.

**TABLE 1:** Leprosy statistics (2020) in South Africa.

Province	Total	Male	Female	Disability grading at diagnosis
Eastern cape	6	2	4	4
Free state	0	-	-	-
Gauteng	8	†	†	
Kwazulu-Natal	2	-	2	1
Limpopo	0	-	-	-
Mpumalanga	0	-	-	-
North West	1	1	-	†
Northern Cape	1	-	-	-
Western Cape	0	-	-	-

**Total**	**17**	**-**	**-**	**-**

Note: Documented Leprosy Incidence in South Africa (updated 1 February 2021), courtesy of Mr. peter Laubscher (The Leprosy Mission)

† , Information not reported.

The diagnosis of leprosy is best made utilising the Ridley and Jopling scale and confirmation based on four components, namely clinical, bacteriologic, immunologic and histopathologic aspect.

### Clinical presentation

There are two basic poles in classifying leprosy, namely lepromatous and tuberculoid leprosy. Patients tend to remain in one form throughout the course of the disease. Cell-mediated immunity of the affected persons largely determines the type of leprosy the specific person will have. Nerve involvement is detected by enlargement of peripheral nerves and appearance of specific skin lesions that are usually associated with loss of sensation.^1,2,3,5,6^

**FIGURE 1:**
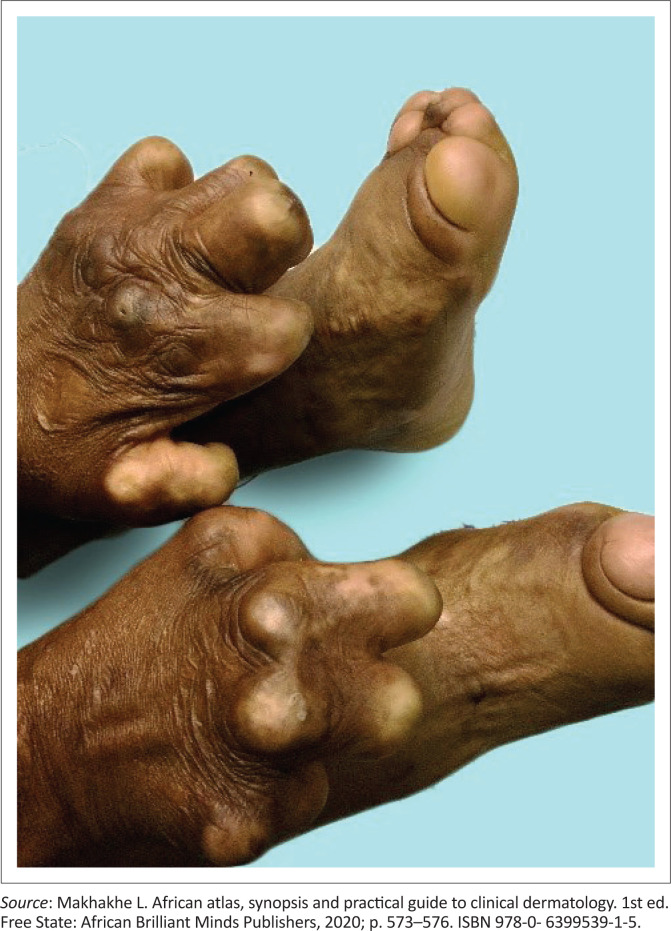
Debilitating complications of end-stage leprosy.

Tuberculoid leprosy results from infection of an individual with high cell-mediated immunity, typified by less than five skin lesions, it can however be more extensive as seen in Figure 1.^7,8^

Hansen’s disease may only involve the nerves; however, it is primarily a disease of peripheral nerves, skin and mucosa, particularly the upper respiratory tract. Skin lesions are usually the first clinical sign noticed and thus the main reasons that it is considered to be a dermatological disease. If left untreated, leprosy may progress causing permanent damage to the skin, the nerves, the limbs and the eyes. Tissue damage may occur because of *M. leprae* infiltration, but most of the damage is secondary to peripheral neuropathy (see Figure 4).

The clinical course is typically insidious, either presenting with numbness before the skin lesions are clinically noticeable and before the peripheral nerves are palpable.

**FIGURE 2:**
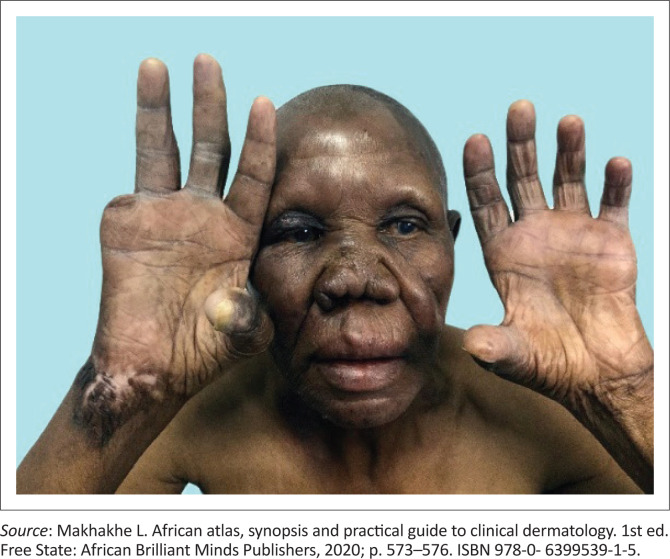
Male patient with lepromateous leprosy, presenting with madarosis (loss of eyebrows), thickened dry skin, nasal congestion and auto-amputation of the little finger on the right hand.

**FIGURE 3:**
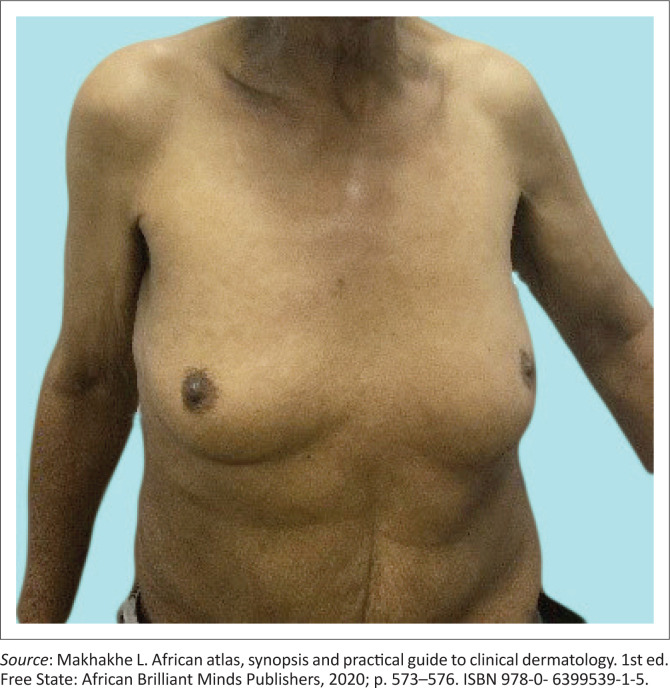
Gynecomastia secondary to testicular infiltration by the bacterium.

Secondary infections, following trauma, after sensory loss may result in tissue loss as bone, cartilage and bone resorption causing auto-amputation of fingers, toes and nasal collapse (see Figures 2 and 3^11^).^9^

The early sensory changes are loss of light touch and temperature. Skin lesions usually begin as one ill-defined, hypopigmented, single patch that can also be erythematous. Furthermore, clinical interrogation may reveal either normal sensation or minimally reduced feeling, often causing disturbances in discerning between hot and cold sensation. A typical lesion of tuberculoid leprosy is anhidrotic (dry skin), with reduced sensation and with secondary alopecia (hair loss). Nerve involvement often appears later, with enlarged and tender peripheral nerves either above or below the skin lesions. Lepromatous leprosy presents as a more severe form of leprosy, it is typically symmetrical, progresses slowly and usually takes longer to treat with higher rates of relapses. Of the two poles, lepromatous leprosy has a severe impact on quality of life (QoL) (see Figures 3, 5, 6, 7 and 8^11^).^3,4,7^

**FIGURE 4:**
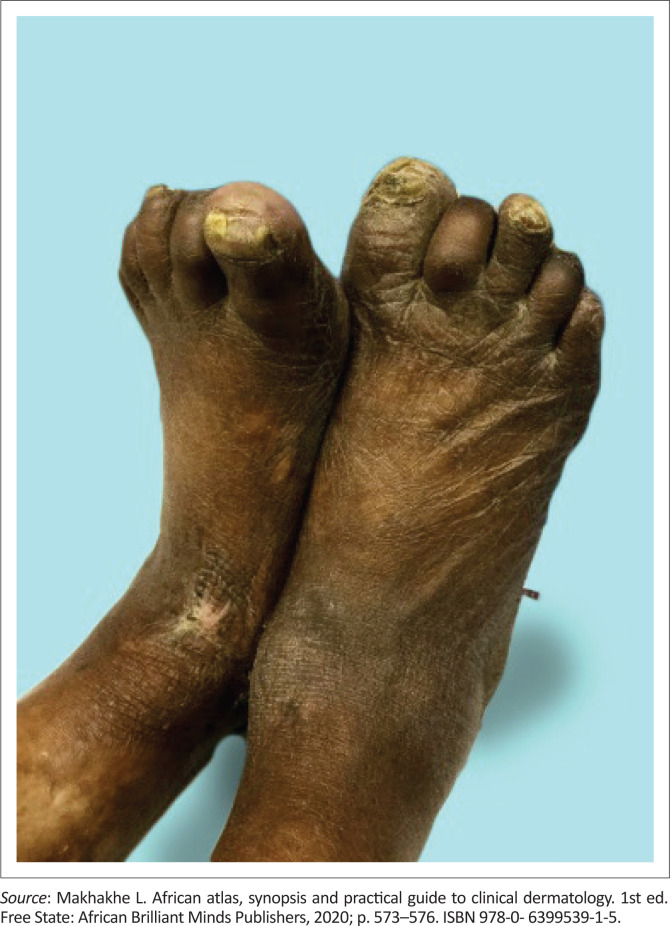
Peripheral neuropathy with secondary nail dystrophy.

**FIGURE 5:**
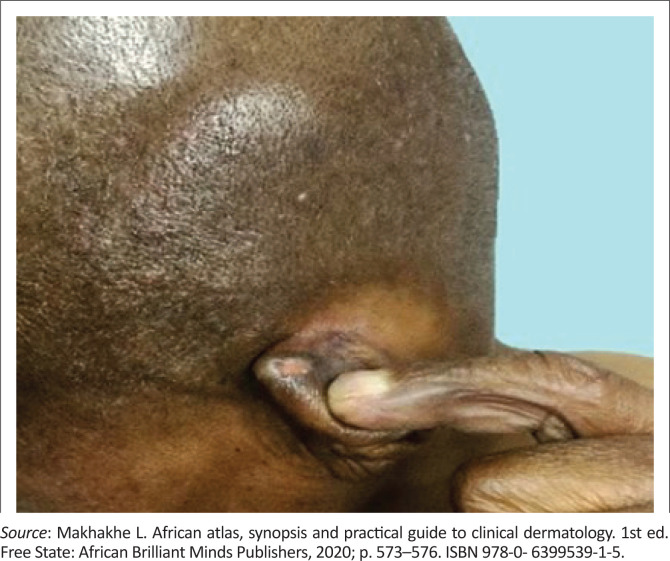
Ulcer on the left ear pinnae, typical area for *M. leprae* as it is cooler than the core body temperature.

**FIGURE 6:**
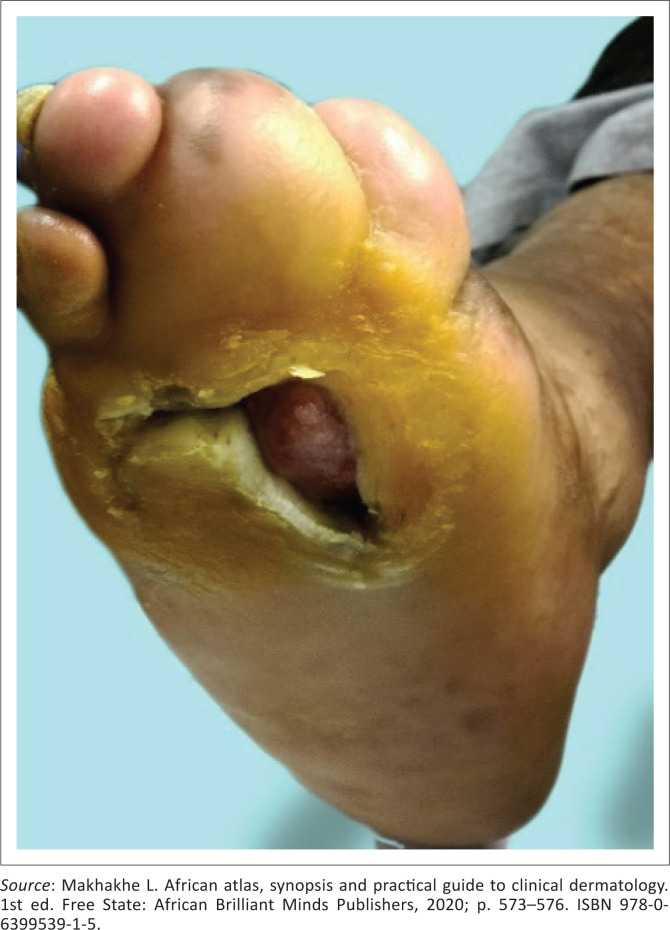
Neuropathic ulcer.

**FIGURE 7:**
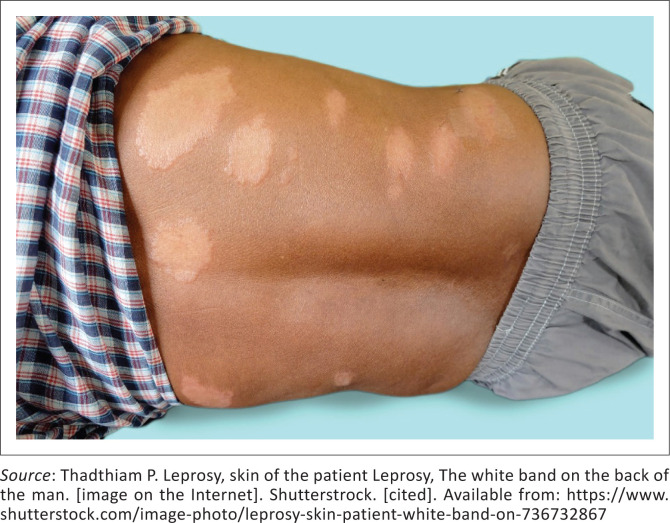
Tuberculoid leprosy

**FIGURE 8:**
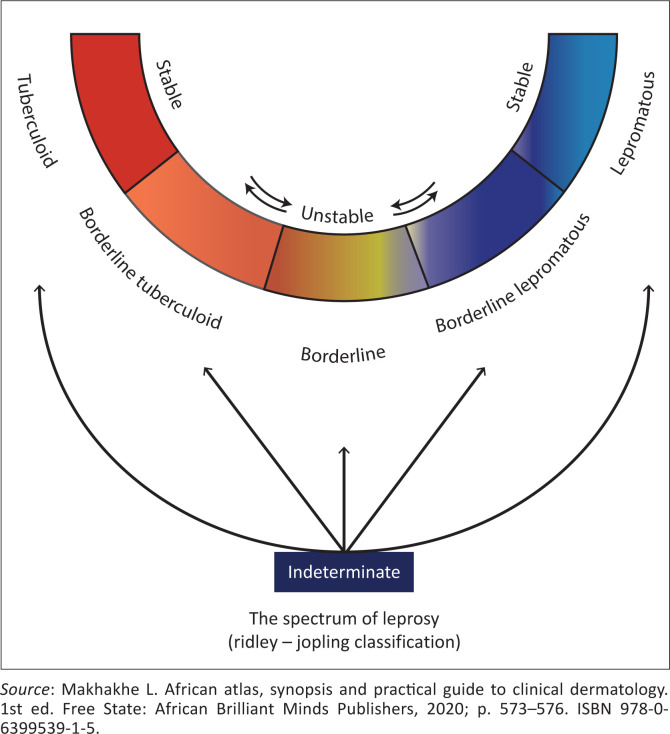
Ridley-Jopling classification.

### Common notable signs in leprosy

The cardinal signs of leprosy are:

- Loss of sensation in a skin lesion
- Enlarged peripheral nerve
- Positive skin smears

Several studies have shown that the presence of antibodies to the *M. leprae*-specific phenolic glycolipid-I (PGL-I) correlates with the bacterial load of a leprosy patient. The majority of multibacillary (MB) patients have high levels of antibodies to PGL-I, whilst in general paucibacillary (PB) patients are seronegative. Studies monitoring the serum antibody levels during treatment further demonstrate this correlation of serum antibodies to PGL-I as indicators of the bacterial load: declining antibody levels during treatment correspond with declining bacterial indices. The observation that increasing levels of antibodies to PGL-I in patients can be associated with the development of relapses also indicates a relation between PGL-I antibody levels and the presence of *M. leprae*.^4,7^

### Management

Management of leprosy includes high index of suspicion, followed by clinical and histological confirmation. Once a diagnosis is confirmed, household members must also be examined whilst the source patient is started on treatment and appropriate monitoring for the full duration of the antibiotic chemotherapy (duration depending on the type of leprosy being treated). Antibiotics include dapsone, rifampicin and clofazimine (see Table 2).^2^ It is also common that leprosy will spontaneously remit in the absence of active treatment.

**TABLE 2:** Common signs of leprosy.

Age group	Drug	Dosage and frequency	Duration	
			MB	PB
Adult	Rifampicin Clofazimine Dapsone	600 mg once a month 300 mg once a month and 50 mg daily 100 mg daily	12 months	6 months
Children (10–14 years)	Rifampicin Clofazimine Dapsone	450 mg once a month 150 mg once a month and 50 mg daily 50 mg daily	12 months	6 months
Children (< 10 years or < 40 kg)	Rifampicin Clofazimine Dapsone	10 mg/kg once a month 6 mg/kg once a month and 1 mg/kg daily 2 mg/kg daily	12 months	6 months

*Source:* Belachew WA, Naafs B. Position statement: Leprosy: Diagnosis, treatment and follow-up. Eur Acad Dermatol Venerol. 2019;33(7):1205–1213. https://doi.org/10.1111/jdv.15569

MB, multibacillary; PB, paucibacillary.

The first known historical treatment that had some effect was chaulmoogra oil, dating back to 600 BC. The effect was minimal in different preparations used, but some success was obtained in PB leprosy.

The first effective antibiotic, intravenous sulfone – promin appeared in 1943, this was followed by an oral derivate called dapsone, which became the standard treatment. Upon the appearance of secondary dapsone resistance in the 1970s together with the ready availability of rifampicin, the use of combined regimens was recommended. However, it was not until 1982 that the WHOs Chemotherapy Study Group recommended the combined use of the two drugs without clofazimine.^3^

In cases of rifampicin resistance, levofloxacin and minocycline can be strongly considered as regimen substitution. South Africa uses WHO’s recommendations in its treatment of leprosy.

### World Health Organization’s recommended treatment for leprosy

#### Reoccurrence

Even after appropriate treatment, there may be a relapse. This can be attributed to a few factors, such as resistance, persisters, re-infections or classification misdiagnosis with subsequent under treatment.

In general, the reoccurrences are sensitive to the original multidrug therapy treatment (MDT). In cases of suspected resistance, PCR test is available. Resistance against clofazimine has not yet been convincingly documented. The main drug in treating multibacillary (MB) leprosy is rifampicin, the same key drug that forms the cornerstone in treating tuberculosis (TB).^4,7,10^

#### Reactions

There are two types of reactions, these usually follow antibiotic therapy.

Type I leprosy reaction (T1R) is also known as reversal reaction, it represents an enhanced cell-mediated immune response to the bacterium. If the reaction occurs as a borderline disease shift towards the lepromatous pole, then they are referred to as downgrading reactions and if this is because of antibiotic commencement, then they are called reversal reactions. Type II leprosy reaction (T2R), also called erythema nodosum leprosum (ENL), these are usually seen in lepromatous leprosy cases believed to be induced by circulating immune-complex mediated factors.

## Conclusion

Bacille Calmette-Guérin (BCG) vaccination remains the best prophylactic therapy in many areas. Rifampicin prophylaxis for close contacts is still highly recommended by WHO, although majority (> 80%) will not contract the infection even without prophylaxis.

**FIGURE 9:**
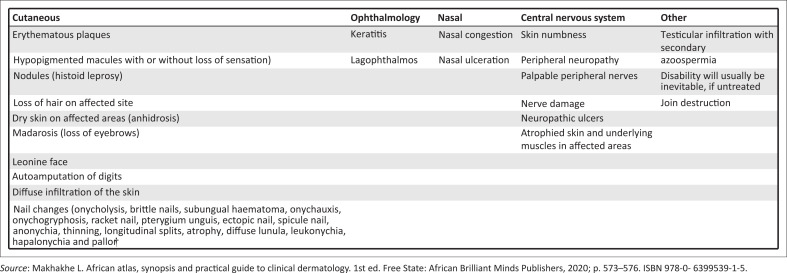
Common signs of leprosy.

A useful tip to keep in mind is that it is rare to have nerve enlargement in other skin diseases. Clinicians need to keep leprosy in mind when confronted with any of the clinical signs as listed in Figure 9, more so when in combination.

Clinicians need to have an index of suspicion so that the infection can be eliminated and ultimately eradicated. Early diagnosis and treatment may lead to less complications and a reduced risk of infection to close contacts. Awareness campaigns and coverage of the disease in both undergraduate and postgraduate programme is important in combating this chronic and often debilitating infection.
